# Transdiagnostic symptom subtypes across autism spectrum disorders and attention deficit hyperactivity disorder: validated by measures of neurocognition and structural connectivity

**DOI:** 10.1186/s12888-022-03734-4

**Published:** 2022-02-09

**Authors:** Manxue Zhang, Yan Huang, Jian Jiao, Danfeng Yuan, Xiao Hu, Pingyuan Yang, Rui Zhang, Liangjian Wen, Mingjing Situ, Jia Cai, Xueli Sun, Kuifang Guo, Xia Huang, Yi Huang

**Affiliations:** 1 Mental Health Center, West China Hospital of Sichuan University, Chengdu, China; 2 University of Electronic Science and Technology of China, Chengdu, China; 3 Brain Research Center, West China Hospital of Sichuan University, Chengdu, China

**Keywords:** Transdiagnostic, Autism Spectrum disorder, Attention deficit hyperactivity disorder, Neurocognition, Diffusion tensor imaging

## Abstract

**Backgrounds:**

Autism spectrum disorder (ASD) and attention-deficit hyperactivity disorder (ADHD) are neurodevelopmental disorders that exhibit within-disorder heterogeneity and cross-disorder phenotypic overlap, thus suggesting that the current disease categories may not fully represent the etiologic essence of the disorders, especially for highly comorbid neurodevelopmental disorders. In this study, we explored the subtypes of a combined sample of ASD and ADHD by integrating measurements of behavior, cognition and brain imaging.

**Methods:**

A total of 164 participants, including 65 with ASD, 47 with ADHD, and 52 controls, were recruited. Unsupervised machine learning with an agglomerative hierarchical clustering algorithm was used to identify transdiagnostic symptom clusters. Neurocognition and brain structural connectivity measurements were used to assess symptom clusters. Mediation analysis was used to explore the relationship between transdiagnostic symptoms, neurocognition and brain structural connectivity.

**Results:**

We identified three symptom clusters that did not fall within the diagnostic boundaries of DSM. External measurements from neurocognition and neuroimaging domains supported distinct profiles, including fine motor function, verbal fluency, and structural connectivity in the corpus callosum between these symptom clusters, highlighting possible biomarkers for ASD and ADHD. Additionally, fine motor function was shown to mediate the relationship between the corpus callosum and perseveration symptoms.

**Conclusions:**

In this transdiagnostic study on ASD and ADHD, we identified three subtypes showing meaningful associations between symptoms, neurocognition and brain white matter structural connectivity. The fine motor function and structural connectivity of corpus callosum might be used as biomarkers for neurodevelopmental disorders with social skill symptoms. The results of this study highlighted the importance of precise phenotyping and further supported the effects of fine motor intervention on ASD and ADHD.

**Supplementary Information:**

The online version contains supplementary material available at 10.1186/s12888-022-03734-4.

## Background

Autism spectrum disorder (ASD) and attention-deficit hyperactivity disorder (ADHD) are neurodevelopmental disorders with symptoms starting to manifest in early childhood [1]. ASD consists of a group of heterogeneous neurodevelopmental disorders characterized by two core symptoms, impairments in social-communication interaction and restrictive and repetitive behaviors [1]. The prevalence of ASD is estimated to be 1–2% [2]. Besides core symptoms, ASD is usually accompanied by other comorbidities [3], among which ADHD is the most common comorbidity [4]. ADHD is characterized by developmentally inappropriate and impairing inattention, motor hyperactivity, and impulsivity [1], with a prevalence of around 5–6% [5].

The symptom-based categorical systems of mental disorders are regarded as the gold standard for diagnosing neurodevelopmental disorders. However, children with ASD or ADHD usually do not entirely fit within the boundaries of a single disorder; instead, they show a mixture of clinical manifestations [6]. For instance, previous studies have suggested that 30–80% of individuals diagnosed with ASD present symptoms of ADHD [7, 8], while 20–60% of ADHD children exhibit autism-like traits [9, 10]. Besides, both disorders show within-disorder heterogeneity and cross-disorder overlap in etiology.

The complex interaction of genetic and environmental factors may contribute to the development of these neuropsychiatric disorders [11]. With a family-aggregation feature [12], ASD and ADHD share candidate genes, such as DAT1 and SLC6A4 [13]. Genetic overlap between disorders may be indicative of an overlapping etiology of ASD and ADHD. It is recommended to study ASD and ADHD simultaneously, and to measure identical behavioral, functional, and structural brain indicators in both patient groups [14]. Given the limitations of using DSM-based phenotypic measurements in genetic analyses, researchers tend to explore pleiotropic risk genes targeting endophenotypes for ASD and ADHD. The Diagnostic Statistical Manual of Mental Disorders 5th edition (DSM-5) allows combined diagnosis of ADHD and ASD, thus acknowledging the possibility of overlapping etiology. The existing diagnosis classification of mental disorders relies on clinicians’ justification of syndromes. Dimensional analysis can be used to divide the symptom mixtures of ASD and ADHD into subtypes.

Deficits in neurocognitive function have been found in both ASD and ADHD [15, 16]. Previous studies have found a set of shared cognitive dysfunctions across ADHD and ASD, such as executive function (EF) and motor function [17]. In order to make timely decisions, motor functions are dependent on strong executive functions, such as cognitive flexibility and inhibition. Moreover, previous studies found that impairments in cognitive flexibility, working memory, ideational fluency, and inhibition were strongly associated with ASD endophenotypes [18, 19]. EF deficits have also been identified in first-degree relatives of ADHD in a wide range of EF tasks [20]. As EF satisfies the criteria of endophenotypes, i.e., unaffected ASD/ADHD first-degree relatives have EF problems, it might be used to disentangle the overlapping phenotype of these two disorders.

Numerous studies have reported abnormal brain structure and function in these two neurodevelopmental disorders [21, 22]. Although studies on microstructural connectivity of ASD and ADHD have revealed inconsistent results, neuroimaging studies indicate shared abnormal small-world properties and corpus callosum white matter deviations in both ASD and ADHD [23–25]. Moreover, several studies have compared ASD and ADHD on brain measurements, but the results remain inconclusive [26–28]. Recently, Tung et al. found that ASD and ADHD share diffuse white matter tract deviations in the corpus callosum, with the prefrontal corpus callosum being more pronounced in ASD. Further correlation analysis revealed that white matter tract deviation was associated with multiple dimensions of psychopathology and cognition including autism symptoms, planning, inhibition, attention, working memory and flexibility [29]. An obstacle in examining the neurobiological etiology of neurodevelopmental disorders is the poor classification of individuals into subtypes by means of existing disease categorical systems based on behavioral observations [30]. Thus, new measurement indicators of neurocognition and neuroimaging, as well as biomarkers of different diagnoses, should be investigated to clarify the homogenous neurobiological mechanisms underlying specific psychopathological phenotypes [31].

Existing studies focusing on ADHD and mood disorders supported the application of the RDoC framework concept in child and adolescent psychiatry [32–34]. Few previous transdiagnostic data-driven studies identified subtypes with different cognitive performance in ASD and ADHD [35–38]. Bathet et al. found that the data-driven classification of EF-related behavioral difficulties had significant individual variation in white matter connections between the prefrontal and anterior cingulate cortices [35]. Vaidya and colleagues found that fronto-parietal engagement was better distinguished by EF subtype than DSM diagnosis, and the impaired subgroup showed lower functional activity of the right inferior parietal lobules [37]. The data-driven EF classification model using a two-stage hybrid machine learning tool showed good robustness, but revealed no “severity” pattern of excessive or insufficient connection among subgroups when using fMRI [36]. This inconsistency might be explained by the fluctuating states of the fMRI study and the indirect measures using Behavior Rating Inventory of Executive Function questionnaire, which estimated by the observations of parents rather than children performance. To the best of our knowledge, no previous study documented valid symptom subtypes in children with ASD and ADHD, nor did they integrate symptom subtypes with multi-model measurement data, which might be useful to address the need to characterize the heterogeneity and overlapping symptoms across diagnostic categories.

Previous studies have reported several main pathways linking neuroimaging or neurocognition with neurodevelopmental disorders. First, neuroimaging abnormalities have been suggested as a candidate endophenotype of ASD and/or ADHD, which directly affects symptoms [21, 22, 39]. However, previous case-control studies showed inconsistencies. In this study, we proposed that symptom subtypes rather than diagnostic categories might help to explore the relationship between neuroimaging and symptoms. Second, EF deficits in both disorders may have similar functional brain origins [40]. Transdiagnostic studies have attempted to explore whether the EF subtypes can map specific functional connections in ASD/ADHD patients, but the findings are inconsistent [36, 37]. The direct relationship between neuroimaging and cognition remains unclear. It is possible that certain cognitive factors moderate the relationship between neuroimaging etiology and symptomatic phenotype. Considering the unclear interaction among cognitive functions, in the present study we selected the parallel multiple mediation model for mediation analysis rather than the serial multiple mediation model.

This study aimed to establish the dimensional symptom subtyping of a combined sample of ASD and ADHD by using a transdiagnostic approach to explore the overlapping neuroimaging and neurocognitive etiology of these two neurodevelopmental disorders. We selected social communication difficulties and inattention/hyperactivity symptoms as the classification inputs for unsupervised machine learning to determine naturally occurring transdiagnostic clusters in a representative combined sample of ASD and ADHD and typically developed children. We hypothesized that children might have symptom clusters, which might not always be within the diagnostic scope, and the symptom clusters could be mapped to specific neurocognition and brain structural connectivity profiles. For instance, abnormalities in the corpus callosum and/or frontoparietal white matter connectivity could be associated with social skill symptoms, which might determine the correlation of EF with ASD/ADHD symptoms. Additionally, we aimed to explore the interrelationships among symptoms, cognition and brain structural connectivity by using a mediation effect model.

## Methods

### Participants and materials

Participants diagnosed with ASD or ADHD were recruited from the West China Hospital outpatient and schools in the local community. Parents/guardians of participants provided the written informed consent for study participation, which was approved by the Medical Ethical Committee of West China Hospital of Sichuan University. Clinical diagnoses were confirmed by experienced child psychiatrists based on DSM-5 and were supported by questionnaires, structured interviews and direct observations. Autism Diagnostic Observation Schedule-General (ADOS-G) [41] and Autism Diagnostic Interview-Revised (ADI-R) [42] were used for ASD diagnosis. The Schedule for Affective Disorders and Schizophrenia for School-age Children-Present and Lifetime Version (K-SADS-PL) [43] Parent Interview was used for ADHD diagnosis. Autism-Spectrum Quotient (AQ) [44] was used for measuring ASD symptoms, and cultural studies had have been investigated in Chinese; this test showed good sensitivity (0.71) and specificity (0.71) [45, 46]. The Swanson, Nolan, and Pelham Rating Scale, IV Version (SNAP-IV) [47] was used to assess ADHD symptoms, and this test has shown good reliability and validity (discriminant accuracy 68.7–75.1%) [47, 48]. Healthy participants recruited from the same community were matched with clinical participants in terms of age and gender. Exclusion criteria for all participants were: co-morbidities with other mental illnesses; estimated IQ score below 70 using the Wechsler Intelligence Scale; genetic syndromes such as Down’s syndrome or Fragile X syndrome; or current use of antipsychotics or stimulants.

All participants obtained K-SADS-PL evaluation. Only patients who were suspected of ASD completed ASD diagnostic evaluation. All parents/guardians of participants completed AQ and SNAP-IV to evaluate symptoms of ASD to ADHD in children, including socialness, mindreading, patterns, attention to details, perseveration (attention switching); inattention (IA), hyperactivity/impulsivity (HI), and oppositional defiant disorder (ODD) symptoms (details of the measures were shown in the supplementary data). After the evaluation, subjects who met the standard were required to perform the cognitive tests: Chinese-Wechsler Intelligence Scale for Children Third Edition (C-WISC-III) [49], Developmental Test of Visual-Motor Integration (Beery VMI) [50]; Purdue Pegboard Test (PPT) [51]; Verbal Fluency (VF) [52]; Cambridge Neuropsychological Test Automated Battery (CANTAB), including Rapid visual information processing, RVP; Stockings of Cambridge, SOC; Spatial Working Memory, SWM; Intra−/Extra-dimensional Set-shift Task, IED. A total of 164 participants obtained the qualified MRI data (ASD, *N* = 65; ADHD, *N* = 47; TDC, *N* = 52). ADHD subtypes included inattention (*N* = 24), hyperactivity/ impulsivity (*N* = 10) and combined types (*N* = 13).

### Data acquisition

All brain imaging was performed on a 3.0-T imaging system (Philips, Achieva, TX, Best, The Netherlands) at the Tibet Chengban Branch of Sichuan University West China Hospital. Three-dimensional T1 images were acquired using a spoiled gradient recalled echoing planar imaging sequence (repetition time, 8.2 milliseconds; echo time, 3.8 milliseconds; flip angle, 7°; slice thickness, 1 mm; field of view, 256 mm × 256 mm; matrix size, 256 × 256; voxel size, 1 × 1 × 1 mm^3^). DTI scans were acquired using a two-dimensional diffusion-weighted echoplanar imaging sequence (repetition time, 10,295 milliseconds; echo time, 91 milliseconds; field of view, 128 × 128 mm; voxel size, 2 × 2 × 2 mm^3^; matrix size, 128 × 128; gradient direction, 32; 75 interleaved, 2-mm; slice thickness, 2 mm; b = 1000 s/mm^2^).

### Image analysis

Data were analyzed using the FSL software (https://fsl.fmrib.ox.ac.uk/fsl/fslwiki/) [53]. Quality control involved eddy current, motion corrections, as well as the removal of non-brain tissues to minimize distortions. Common DTI metrics consisted of fractional anisotropy (FA), mean diffusivity (MD), radial diffusivity (RD), and axial diffusivity (AD). FA, the fraction of anisotropic diffusion, was the most commonly used metrics. The breakdown in white matter integrity typically resulted in a lower FA [54]. For image analyses Tract-Based Spatial Statistics [55] was conducted using FA as DTI metrics in this study. Mean FA skeleton was created by aligning each participant’s FA image to the Montreal Neurological Institute (MNI) 152 space template. The FA threshold was set at 0.2 to suppress areas of low fractional anisotropy. Threshold of statistical maps was set to *p* < 0.05, with TFCE FWE fully corrected for multiple comparisons.

### Statistical analysis

Statistical analyses were conducted using Jupyter, scikit-learn, NumPy, SciPy, and matplotlib packages (Python, version 3.6.2). Unsupervised machine learning was adopted to classify children into clusters. Z scores were applied for cognitive profiles measured in different metrological units. The inverse values were inverted to facilitate the creation of a numerical matrix, e.g., SWM and IED scores. Eight subscale Z-scores of AQ and SNAP-IV (Socialness, Mindreading, Patterns, Attention to Details, Perseveration, Inattention, Hyperactivity and ODD) for all 164 children were input into the agglomerative hierarchical clustering to generate clusters. With stability to generate clusters even in small samples, agglomerative hierarchical clustering was performed. The optimal number of clusters was determined using two standard methods including the gap statistic [56] and the dendrogram. Cluster centers were plotted from 1000 repeated subsamples to assess the clustering solution’s robustness. To provide an efficient and more easily visualized method to describe cluster differences, linear discriminant analysis (LDA) was conducted. These discriminant functions were also used to test classification accuracy. Furthermore, symptoms were chosen as the classification variable to summarize the relationship between the symptom composite variables.

One-way analysis of variance (ANOVA) was used to compare ASD, ADHD and control groups, followed by Bonferroni multiple-comparisons test. We evaluated how the clusters differentiated on the symptoms, neurocognition, and microstructural connectivity by ANOVA analysis with post-hoc Bonferroni correction. To provide a complementary diagnostic frame of reference for interpretation, the symptom clusters were mapped onto the DSM diagnostic categories. Pearson correlation was used to explore the association among FA values, cognitions, and clinical symptoms. Statistical analyses were performed using SPSS (IBM, 26.0 version).

Finally, a mediating effect model was constructed using Amos version 21.0 to examine how cognition affected the relationship between brain structural connectivity and the symptoms. The following indexes were used to check model fit [57]: a) root mean square error of approximation (RMSEA), with a value of 0.08 or less reflecting a reasonable fit; b) comparative fit index (CFI), with a value of 0.90 and higher indicating a good fit. The number of bootstrap samples for bias-corrected bootstrap confidence intervals was 10,000.

## Results

### Demographic characteristics of participants

All participants were Han Chinese and right-handed. There were no significant differences among the three groups (ASD, ADHD, and control groups) with respect to age, sex, maternal infection during pregnancy, birth, neonatal diseases, and parental education levels (all *P* > 0.05). Although there were differences in IQ among groups (F = 15.20, *p* < 0.001, post-hoc t-tests, ASD < ADHD/TDC), all participants had a IQ > 70 (Table 1) (Table 1 was placed at the end of the document text). As seen in Table 1, there were significant differences in AQ and SNAP-IV (AQ: F = 16.254, *p* < 0.001, post-hoc t-tests, ASD > ADHD/TDC; SNAP-IV: F = 31.558, p < 0.001, post-hoc t-tests, ADHD>ASD/TDC). Considering the imbalanced IQ distribution among groups, IQ was set as a confounder in the ANOVA analysis. Results showed differences among groups in PPT, VP, MC, VF-se, SOC, and SWM. Post hoc t tests revealed differences between ASD and ADHD in PPT, MC, and SWM (details in Table 1).

**Table 1:** General demographic characteristics of participants

	ASD [1] *N* = 65	ADHD [2] *N* = 47	TDC [3] *N* = 52	F/χ^2^	p	Post-hoc
Sex (Woman /Man)	10/55	6/41	9/43	0.396	0.821	–
age	9.27(3.56)	8.68(2.26)	8.48(2.42)	1.187	0.308	–
Min	3	6	5			
Max	17	16	16			
IQ	92.92(17.45)	109.2(16.99)	105.49(15.12)	15.206	< 0.001***	1 < 2/3
Min	70	73	70			
Max	128	143	135			
AQ	44.00(13.85)	32.64(9.91)	33.56(11.13)	16.254	< 0.001***	1 > 2/3
SNAP-IV	35.75(10.84)	41.51(9.61)	25.52(9.93)	31.558	< 0.001***	2 > 1/3
Socialness	18.60(6.59)	11.79(5.94)	11.81(5.67)	24.17	< 0.001***	1 > 2/3
Mindreading	12.29(6.15)	9.89(5.07)	8.25(5.04)	7.97	0.001**	1 > 3
Patterns	6.12(2.85)	6.34(2.53)	6.08(2.57)	0.14	0.872	–
Details	6.31(2.33)	5.79(2.30)	5.44(2.59)	1.92	0.15	–
Perseveration	10.11(3.79)	8.66(2.25)	8.10(3.01)	6.35	0.002**	1 > 3
IA	14.57(4.40)	18.38(3.25)	12.77(4.46)	23.72	< 0.001***	2 > 1/3
HI	10.98(4.17)	14.17(4.91)	9.13(5.24)	14.17	< 0.001***	2 > 1/3
ODD	8.18(4.13)	11.94(4.83)	8.25(4.79)	11.22	< 0.001***	2 > 1/3
ADOS-G	18.04(5.28)	–	–	–	–	–
ADI-R	41.66(16.71)	–	–	–	–	–
PPT-hands	30.98 (9.93)	36.99 (8.01)	36.17 (6.65)	5.244	0.006**	1 < 2/3
PPT-assemble	17.60 (7.25)	22.00 (7.61)	25.61(6.69)	13.184	< 0.001***	1 < 2 < 3
VP	103.06 (15.99)	108.04 (11.02)	113.67(11.48)	5.642	0.004**	1 < 3
MC	94.75 (18.04)	97.15 (17.88)	106.52 (14.06)	5.943	0.003**	1/2 < 3
VMI	96.06 (19.78)	99.57 (16.17)	105.48 (11.86)	2.874	0.59	1 < 3
VF se	14.14 (5.06)	15.13 (4.74)	16.85 (5.61)	3.211	0.043 *	1 < 3
VF ph	4.75 (2.92)	4.00 (2.54)	5.02 (3.04)	1.868	0.158	–
RVP	0.89 (0.09)	0.90 (0.08)	0.91 (0.07)	0.533	0.588	–
SOC	5.65 (3.17)	4.81(2.35)	6.27(2.42)	4.607	0.011*	2 < 3
SWM	−54.58(25.12)	−51.77 (26.13)	−34.73 (27.39)	8.174	< 0.001***	1/2 < 3
IED	−47.23 (23.09)	−48.09 (20.16)	−39.62 (25.50)	2.267	0.107	–

Note: One-Way ANOVA with Bonferroni post-hoc test. *p < 0.05, **p < 0.01, ***p < 0.001. IQ: intelligence quotient. *AQ* Autism-Spectrum Quotient total scores; *SNAP-IV* Swanson Nolan and Pelham, Version IV Scale total scores; socialness: AQ socialness subscale, mindreading: AQ mindreading subscale, patterns: AQ patterns subscale, details: AQ attention to details, perseveration: AQ attention switching/perseveration subscale. *IA: SNAP-IV* inattention subscale, *HI: SNAP-IV* hyperactivity/impulsivity, *ODD: SNAP-IV* oppositional defiant disorder subscale; *PPT* Purdue Pegbord Test measuring fine motor function, *VP* visual perception, *MC* motor coordination, *VMI* visual motor integration, *VF-se* semantic verbal fluency, *VF-ph* phoneny verbal fluency, *RVP* rapid visual information processing, measure for sustain attention, *SOC* stockings of cambridge, measure for planning, *SWM* spatial working memory, measuring working memory, *IED* Intra−/Extra-dimensional Set-shift Task, measuring regular

### Unsupervised machine learning algorithms

**Fig. 1:**
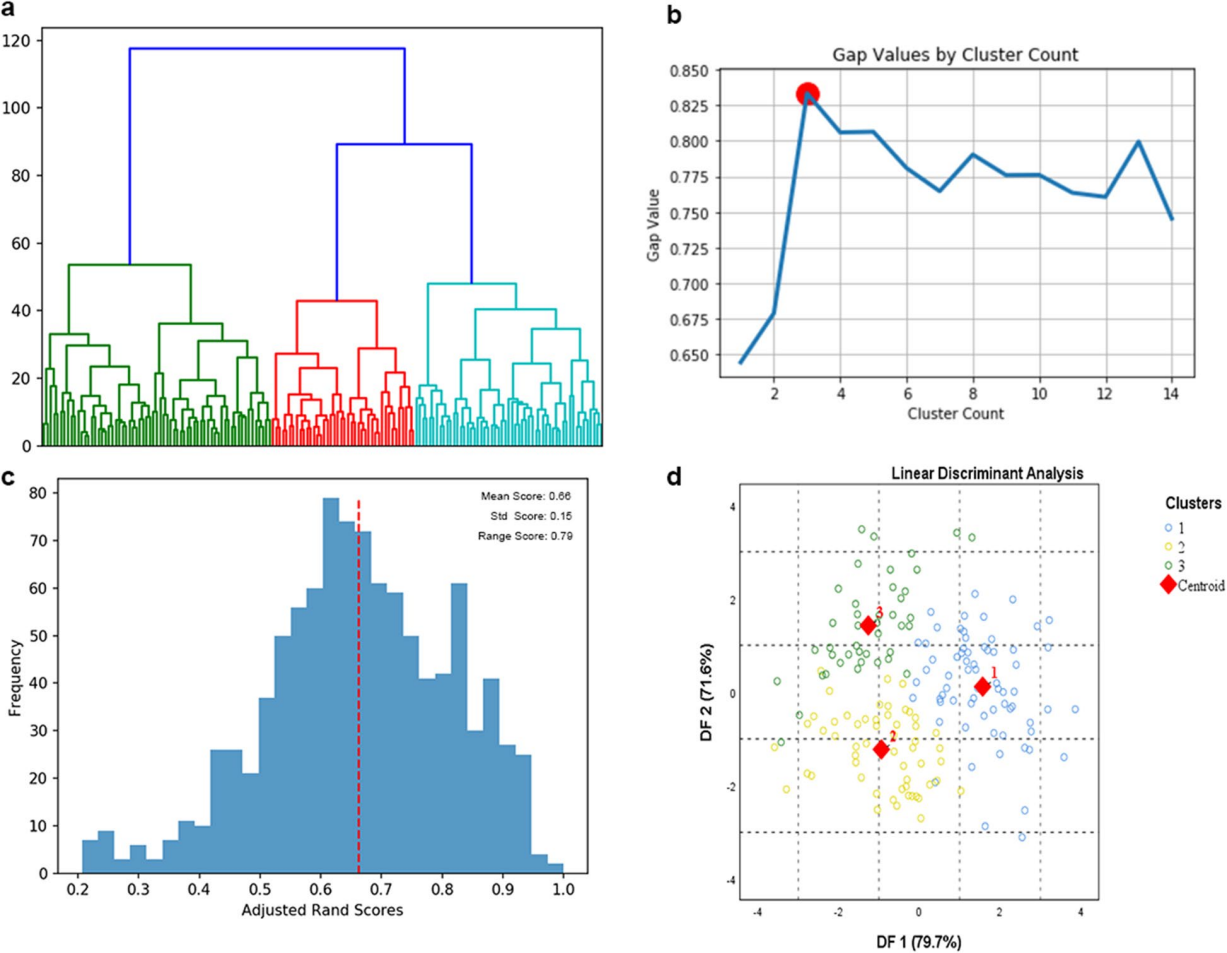
The characters of the unsupervised classification analysis. **a** The dendrogram for hierarchical clustering. The y-axis represents the distance between clusters. Colors represent the three-cluster solution chosen. **b** The gap statistic, calculated for cluster counts from 1 to 15. The line charts show the optimal cluster number based on the gap statistic values and the red circles indicate the optimal cluster number. In this context, the most optimal cluster number was 3. **c** The distribution of adjusted Rand scores visualized in a histogram. To evaluate the stability of the clustering solution, we repeated the clustering analysis in randomly selected subsamples (each containing 80% of the subjects) for 1000 times. In each of the 1000 subsamples, the remaining 20% subjects left out were assigned to clusters using linear discriminant analysis classifiers. These two samples combined to form a complete cluster solution. We then tested the stability of clustering over the 1000 subsamples by calculating an adjusted Rand score, which represent the similarity between each clustering solution compared to the original clustering solution. The average adjusted Rand score was 0.66 (min 0.21, max 0.79). **d**. Linear discriminant analysis. Linear discriminant analysis is a supervised classification method that constructs a predictive model to evaluate the group membership, based on the theory of Bayes formula. Thus, these new variables are called discriminants functions (DF’s) that provide the best discrimination between the groups

The gap statistics and the dendrogram results showed that the optimal number of clusters was 3 (Fig. 1a, b). The robustness of clustering over the 1000 subsamples demonstrated good stability: the average adjusted Rand score was 0.66 (min 0.21, max 0.79) (Fig. 1c). The linear discriminant analysis (LDA) of the eight symptom dimensions showed that the classification accuracy in predicting clusters was 92% (Fig. 1d, Table 2). The selection of the optimal clustering was determined with various statistical techniques as guides, such as Silhouette Score (0.23); Calinski-Harabaz Index(51.57); Dunn Validity Index(0.21).

**Table 2:** Classification accuracy to predict clusters

	Predicted cluster members			
Original	1	2	3	Total
Cluster 1	62(92.5%)	2(3%)	3(4.5%)	67(100%)
Cluster 2	0	52(94.5%)	3(5.5%)	55(100%)
Cluster 3	0	2(4.8%)	40(95.2%)	42(100%)

Note: The classification accuracy was 92%

**Fig. 2:**
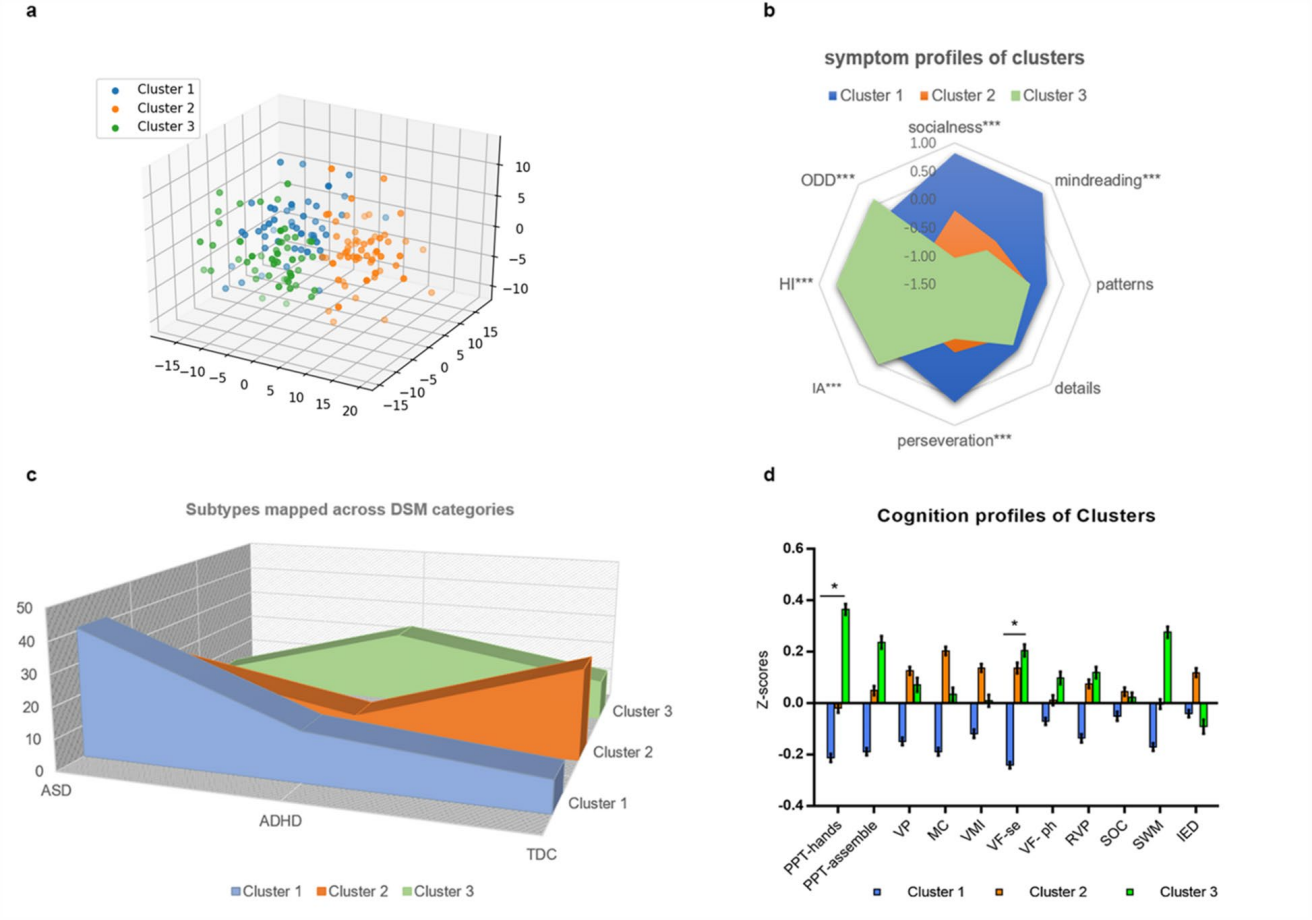
Symptom clusters with validations. *p < 0.05, ***p < 0.001. **a** Visual demonstration of clustering in 3-dimensional space. The original eight symptom dimensions were reduced to 3 dimensions by principal component analysis in this figure for visualization. Each component is a spatial dimension on the x-axis, y-axis, and z-axis. **b** Comparative radar plots of the symptoms in the three clusters. ***p < 0.001, Symptom Z scores on the y axis. Socialness: AQ socialness subscale, mindreading: AQ mindreading subscale, patterns: AQ patterns subscale, details: AQ attention to details, perseveration: AQ attention switching/perseveration subscale. IA: SNAP-IV inattention subscale, HI: SNAP-IV hyperactivity/impulsivity, ODD: SNAP-IV oppositional defiant disorder subscale. **c** Subtypes mapped across DSM diagnostic categories. Each slice of the area chart shows the numbers of children in that DSM diagnostic category fell into a specific symptom cluster. **d** Cognition profiles in three clusters. All values are expressed in standardized units to facilitate interpretation of profiles across measures. Error bars represent 1 standard error of mean (SEM). PPT: Purdue Pegbord Test measuring fine motor function, VP: visual perception, MC: motor coordination, VMI: visual motor integration, VF-se: semantic verbal fluency, VF-ph: phoneny verbal fluency, RVP: rapid visual information processing, measure for sustain attention, SOC: stockings of cambridge, measure for planning, SWM: spatial working memory, measuring working memory, IED: Intra−/Extra-dimensional Set-shift Task, measuring regular

The unsupervised machine learning algorithm identified a 3-cluster solution; characterized either by social impairment symptoms (C1, *n* = 67), relatively normal children (C2, *n* = 55), or inattention/hyperactivity/impulsivity symptoms (C3, *n* = 42) (Fig. 2a). Clusters did not significantly differ when considering age, sex, or IQ (Table S1). ANOVA results showed symptoms differences among subtypes, except in patterns and attention to detail symptoms (Fig. 2b, Table S1). As indicated in Table S2, the symptoms showed favorable discriminant function in separating the three clusters. The principal components analysis for the three symptom subtypes showed two mean PCs, C1 distributed in the PC1(social skill symptoms), C3 distributed in the PC2(IA/HI/ODD symptoms), while C2 distributed both in the two PCs (Fig. S1).

### Validation of clusters differences via external measurements

#### Comparison with conventional diagnostic categories

Frequency distribution of DSM diagnostic categories in different clusters showed that clusters included children from all diagnostic groups (× 2 = 47.28, *p* < 0.001), which suggested that clusters did not represent the original diagnosis (Table S3). As shown in Fig. 2c, children with traditional diagnoses of ASD and ADHD were distributed across all three clusters.

#### Neurocognition variations among symptom clusters

Clusters were expressed in a differential profile of cognitive performance (Fig. 2d). Fine motor function, as measured by PPT hands test, was found to be worse in C1 than in C3 (Z-scores were − 0.213 in C1, − 0.019 in C2, and 0.365 in C3; F2,164 = 4.51; *p* = 0.0124, ηp2 = 0.053; post-hoc test showed C1 < C3). Semantic verbal fluency in C1 was worse than that in C3 (Z-scores were − 0.213 for children in C1, − 0.019 in C2, and 0.365 in C3; F2,164 = 4.51; *p* = 0.0341, ηp2 = 0.041; post-hoc test showed C1 < C3). There were no significant differences in other cognitions among clusters (*p* > 0.05, Table 3).

**Table 3:** Cognition profiles among three clusters

	Cluster 1	Cluster 2	Cluster 3	F	p	Post-hoc
IQ	98.57(18.07)	101.99(17.02)	105.82(18.66)	2.15	0.1203	–
Min	70	70	70			
Max	135	143	135			
PPT-hands	32.47 (9.30)	34.18 (8.42)	37.58 (7.87)	4.51	0.0124 *	C1 < C3
PPT-assemble	19.91(7.63)	21.79 (7.82)	23.27(8.18)	2.48	0.0871	–
VP	105.78 (14.13)	109.62 (11.83)	108.86(16.14)	1.29	0.2787	–
MC	95.87 (17.60)	102.73 (15.88)	99.79 (18.73)	2.40	0.0940	–
VMI	98.04 (19.01)	102.38 (14.41)	100.21 (16.51)	0.99	0.3734	–
VF se	14.01 (4.42)	16.00 (6.00)	16.36 (5.12)	3.45	0.0341 *	C1 < C3
VF ph	4.42 (2.65)	4.65 (3.08)	4.90 (2.95)	0.38	0.6879	–
RVP	0.89 (0.09)	0.91 (0.07)	0.91 (0.07)	1.08	0.3416	–
SOC	5.46 (3.31)	5.73(2.48)	5.67(2.16)	0.15	0.8598	–
SWM	−52.15(27.57)	−47.58 (28.25)	−39.90 (24.95)	2.62	0.0756	–
IED	−46.00 (21.98)	−42.29 (22.10)	− 47.19 (26.75)	0.62	0.5408	–

Note: *p < 0.05, *IQ* intelligence quotient, *PPT* Purdue Pegbord Test measuring fine motor function, *VP* visual perception, *MC* motor coordination, *VMI* visual motor integration, *VF-se* semantic verbal fluency, *VF-ph* phoneny verbal fluency, *RVP* rapid visual information processing, measure for sustain attention, *SOC* stockings of cambridge, measure for planning, *SWM* spatial working memory, measuring working memory, *IED* Intra−/Extra-dimensional Set-shift Task, measuring regular

#### Microstructural connectivity differences among clusters

**Fig. 3:**
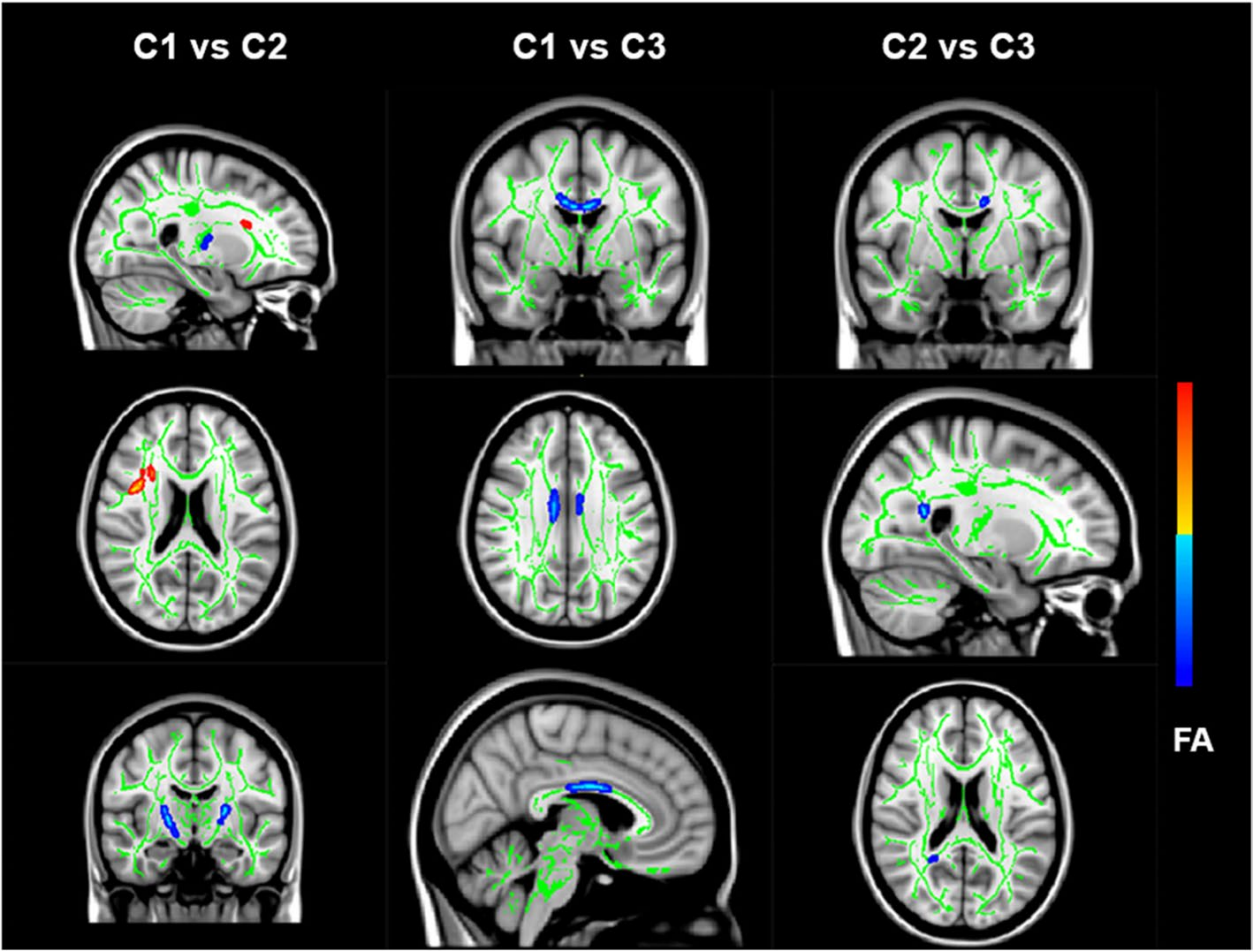
Brain white matter structural differences among C1, C2, C3 clusters on TBSS analysis. TBSS results within the International Consortium for Brain Mapping DTI-81 atlas are reported and labeled accordingly (TFCE FWE corrected, p < 0.05). red symbols FA values increase, blue symbols FA values decrease. First line: C1 < C2 (blue): Cerebral peduncle R; Posterior limb of internal capsule L; Posterior limb of internal capsule R; C1 > C2(red): Anterior corona radiata R. Second line: C1 < C3 (blue): Body of corpus callosum. Third line: C2 < C3 (blue): Body of corpus callosum; Splenium of corpus callosum

In the pairwise comparisons of voxel-wise analyzed by TBSS, we found lower FA of the corpus callosum in C1. Lower FA was found in the C1 compared with the C2 as voxels located along the corpus callosum, right cerebral peduncle, and posterior limb of the internal capsule; C1 had lower FA in the body of the corpus callosum than C3 (Fig. 3).

### Symptoms associated with microstructural connectivity and cognitions

**Fig. 4:**
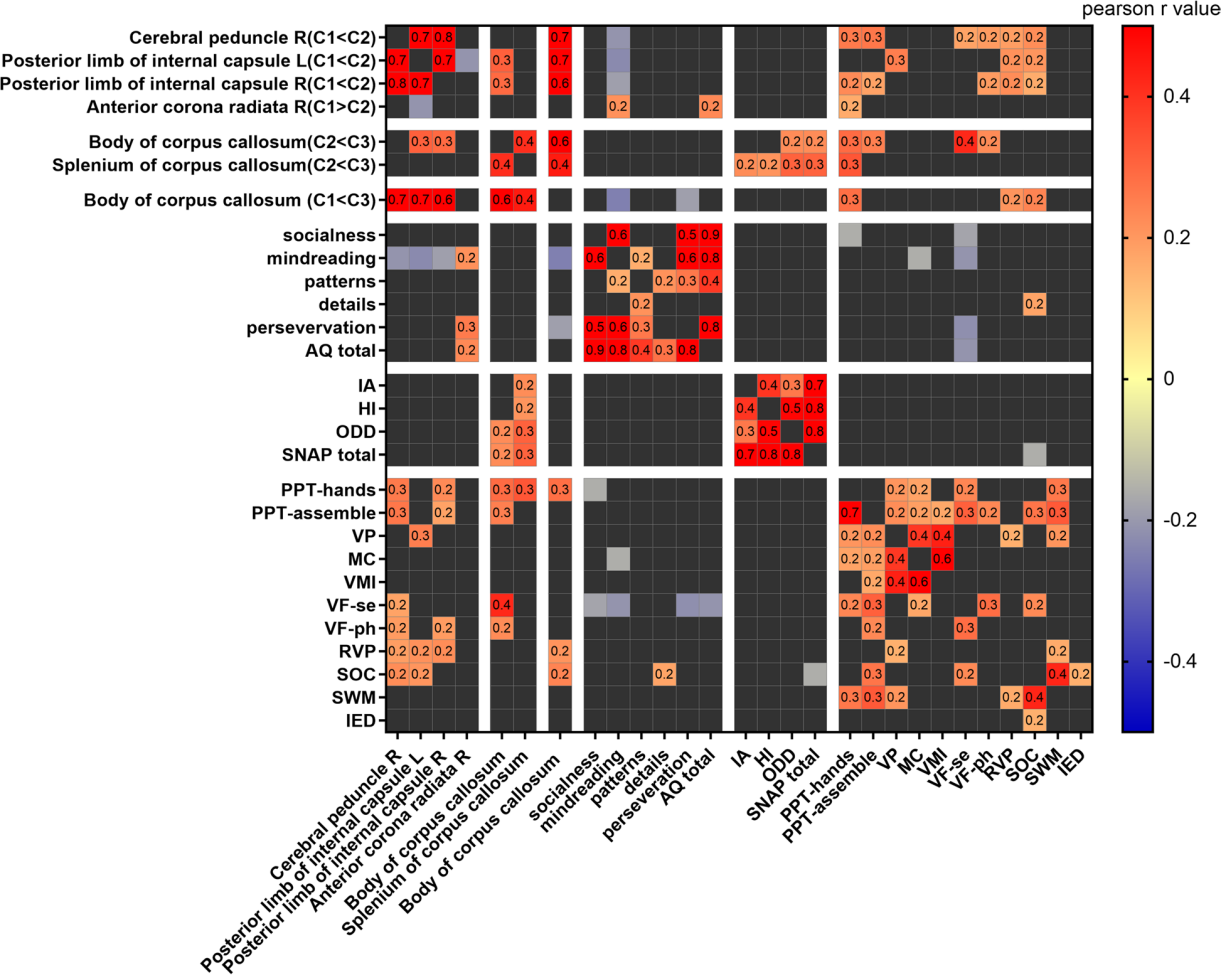
The correlation heatmap among symptoms, cognitions and microstructural connectivity. The gradient color barcode at the top-right indicates the minimum value in blue and the maximum in red. The correlation value shown in the cells were calculated by pearson correlation analysis

Pearson correlation analysis revealed a positive correlation between splenium of corpus callosum (C2 < C3), IA/HI symptoms (r = 0.222, *p* = 0.03; r = 0.209, *p* = 0.04; respectively), cognitive skill-like motor function and verbal fluency. The body of the corpus callosum (FA value C1 < C3) was found to be linked with perseveration symptoms (r = − 0.192, *p* = 0.04) and PPT-hands (r = 0.28, *p* = 0.003). The FA of the internal capsule (C1 < C2) was correlated with mindreading symptoms (r = − 0.231, *p* = 0.01), and was slightly correlated with fine motor function (r = 0.250, p = 0.01), planning (r = 0.206, *p* = 0.02), sustain attention (r = 0.214, p = 0.02) (Fig. 4). The PPT-hands test and semantic verbal fluency were negatively correlated with socialness symptoms (respectively = − 0.158, *p* = 0.04; r = − 0.184, p = 0.02), the SOC was negatively correlated with SNAP total (r = − 0.159, p = 0.04) (Fig. 4).

### Fine motor function mediating the relationship of the body of corpus callosum and perseveration symptoms

**Fig. 5:**
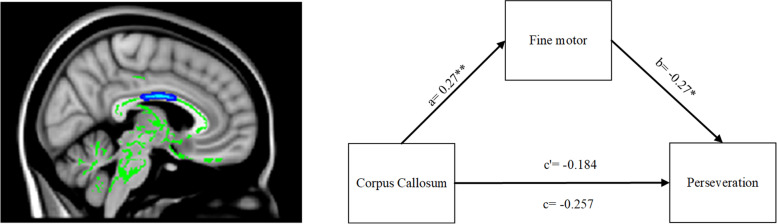
Fine motor function mediating between the brain structural connectivity and perseveration symptoms. *p < 0.05, **p < 0.01; Fine motor: measured by purdue pegboard hands subtest; Corpus Callosum: the FA value of the body of the corpus callosum, which was the structural difference between C1 and C3(featured in the left picture); Perseveration: measured by the AQ-perseveration subscale. χ2 = 10.803, df = 10, χ2 /df = 1.08, root mean square error of approximation, RMSEA = 0.027; comparative fit index, CFI = 0.990; a, b, c variables’ value represented the standard coefficient

Across subtypes C1 and C3, PPT-hands were found to be linked with perseveration symptoms (*r* = − 0.22, *p* = 0.022) and the corpus callosum (*r* = 0.28, *p* = 0.003), the corpus callosum was correlated with the perseveration symptoms (*r* = − 0.19, *p* = 0.036). Moreover, the mediating effect model among the corpus callosum brain structural connectivity, fine motor function, and perseveration showed adequate goodness of fit (χ2 = 10.803, df = 10, χ2 /df = 1.08, root mean square error of approximation, RMSEA = 0.027; comparative fit index, CFI = 0.990). The corpus callosum structural connectivity predicted the perseveration symptoms (total effect, c = − 0.257), while a direct path from corpus callosum to perseveration was not significant (direct effect, c’ = − 0.184, *p* > 0.05) (Fig. 5). These data suggested that fine motor function had a total mediating effect on the association between the corpus callosum and perseveration symptoms.

## Discussion

The present study demonstrated a transdiagnostic approach to identify subtypes of a combined sample of ASD and ADHD defined by distinct profiles of symptoms, cognition and brain structural connectivity. Three symptom clusters (C1: social impairment symptoms, C2: relatively normal children, C3: inattention/hyperactivity/impulsivity symptoms) were determined by using agglomerative hierarchical clustering, which were not mapped onto the DSM categories. This study indicated white matter variations among subtypes. Our results also revealed that the C1 subtype was distinct from the C3 subtype in fine motor dysfunction and verbal fluency. Neuroimage-neurocognition-transdiagnostic symptoms interactions were investigated via correlation analysis, and the results revealed an association between WM structural connectivity, motor functions/verbal fluency/EFs, and ASD/ADHD symptoms. To the best of our knowledge, this was the first study to identify fine motor function as a mediator for the corpus callosum structural connectivity and perseveration symptoms. Our findings provided an opportunity for a more biomarker-based approach for disease subtyping, evidenced on precise phenotypes of a large category of neurodevelopmental disorders including ASD and ADHD.

C1 subtype had fine motor dysfunction, which was consistent with previous within-diagnosis research in ASD and ADHD [58, 59]. Our study also indicated that the fine motor function was associated with social symptoms. Although fine motor dysfunction was a non-diagnostic symptom of ASD, the impaired fine motor function was linked with social deficit and repetitive behaviors in ASD [58]. Previous studies suggested that fine motor skills were connected with social skill development in healthy children [60–62]. Moreover, fMRI study in ASD indicated that the abnormal functional connectivity of the sensorimotor system continued to the middle-age and elderly adulthood [63]. These results suggested that fine motor function might be involved in the pathogenesis and progression of social skill impairment in children with ASD and ADHD.

Our study also indicated dysfunction of verbal fluency in the C1 subtype, thus suggesting that deficit in verbal fluency was associated with impairment in social skills in ASD and ADHD. This result was partly in line with previous studies, which reported that ASD is associated with various semantic abnormalities [64]. Furthermore, our data showed that children with ADHD exhibited more impairment in verbal fluency than those with IQ-matched ASD [65]. To sum up, this study provided further evidence in support of verbal fluency as the neurocognitive marker of neurodevelopment disorders such as ASD and ADHD.

As for other cognition profiles of the three clusters, we failed to identify any differences in EF among the three symptom subtypes, which was somewhat surprising considering that specific EFs were shown to be linked with ASD and ADHD in a number of studies [66, 67]. In addition, one data-driven study found that transdiagnostic EF subtyping could not be attributed to symptom presentation in ASD and ADHD [36]. This indicated that EF was independent variable, not specific to categorize diagnostic symptoms. Further studies with broader EFs and a larger sample size are needed to clarify the relationship between cognitive subtypes and the symptom severity of neurodevelopment disorders.

In the present study, three subtypes showed distinct neuroimaging profiles. Firstly, when comparing C1 with C3, C1 was characterized by decreased structural connectivity in the body of the corpus callosum. The FA values showed differences in the body/splenium of the corpus callosum when comparing C2 with C3. The symptom dimensional analysis showed that the corpus callosum (C1 vs. C3) was associated with mindreading and perseveration symptoms, while the corpus callosum (C2 vs. C3) was associated with ADHD symptoms. This suggested that the corpus callosum played a role in the overlapping of ASD-ADHD spectrum disorders. Consistent with our findings, a dimensional analysis showed that ASD symptom severity and inattention symptoms were associated with FA of the corpus callosum across ASD and ADHD [26]. However, previous studies also showed that ASD had a lower FA of the corpus callosum compared to ADHD [26, 27], suggesting that the white matter morphology in the corpus callosum might be used to differentiate ASD from ADHD. The corpus callosum is the largest commissural tract in the human brain, connecting homologous regions of the two cerebral hemispheres [68], and is associated with social function [69], motor function [70], sustain attention and EF [71]. Lower FA in the corpus callosum in imaging studies indicated decreased directional organization of white matter microstructure, which might be due to changes in myelination, axonal density and axonal degeneration [72]. Combined with our findings, different white matter structural connections of the corpus callosum could partially explain the neural mechanisms underlying behavioral differences between ASD and ADHD.

We found decreased FA within the internal capsule in the C1 subtype compared with C2, and the internal capsule was correlated with mindreading symptoms. The result was aligned with previous studies that indicated social communication was associated with internal capsules in ASD [73, 74]. Additionally, although both C1 and C2 subtypes showed impaired social skills, the former was more severely affected. This suggested that internal capsule is a neuroimaging feature of social skill impairment among ASD and ADHD. Both in C1 and C2, children might have similar internal capsule abnormalities to varying degrees. The difference might be explained by cognitive types such as motor function and EFs. Further structural equation models with larger samples are necessary to validate this hypothesis.

The existence of three independent symptom clusters was statistically justified, which showed distinct symptom profiles cutting across DSM diagnostic categories. Yet, the discriminant analysis results showed good predictive accuracy (92%) of the symptom dimensions, thus indicating that the clustering analysis result was reliable. There was no difference in two typical autism repetitive and stereotyped behaviors (RBBs) symptoms: patterns and attention to detail, which might be explained by the overlapping phenotypic characteristics of ADHD and ASD [9, 10, 75]. Stereotypical and repetitive behaviors were also common even in healthy infants and toddlers. It indicated that RBB’s symptom dimension is still a group of syndromes, including different behavioral dimensions (such as abnormal sensory experience, repetitive behavior, monotonous hobbies), and may still have great heterogeneity. This suggested that it might be useful to adopt dimensional analysis to explore the etiology of neurodevelopmental disorders.

**Fig. 6:**
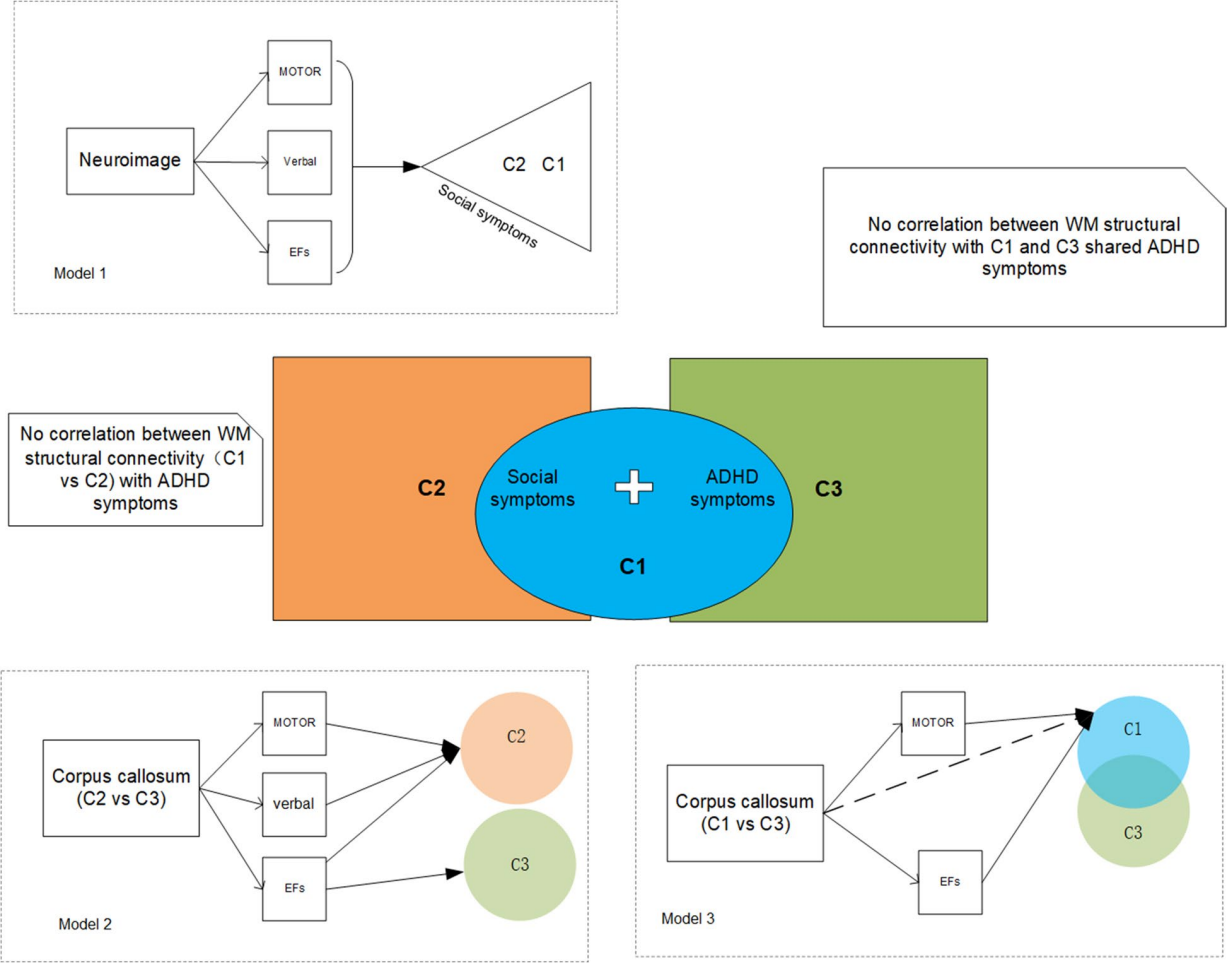
The hypothesis of the neuroimage- cognitions- ASD/ADHD symptoms. Note:Model 1 indicating that C1 and C2 are, in fact, different manifestations of the same overarching disorder with shared neuroimaging etiology. The manifestations range from C2 with few social symptoms to C1 as the most severe subtype characterized by severe social and ADHD problems. Model 2 proposed that cognitive endophenotypic traits mediate between C2/C3 subtypes distinct social symptoms and certain neuroimage abnormality. Model 3 demonstrated that the cognitive endophenotype is useful for mediating the associations between WM structural abnormality and ASD-ADHD spectrum. Differentiating between Models 2 and 3 is weather cognitive endophenotype and symptom are overlapping in subtypes

The present study indicated several links among WM structural connectivity, cognitions, and ASD/ADHD symptoms. Nevertheless, determining how neuroimage is linked to ASD/ADHD symptoms and how cognition may be candidate endophenotypes for neurodevelopment disorders, including ASD and ADHD, is challenging. A simplified schematic form for neuroimaging-cognition endophenotypes- symptoms of ASD and ADHD are depicted in Fig. 6**.** As we proposed we have found associations between the corpus callosum with sensorimotor functions, which is consistent with previous studies [69, 74]. Fine motor function was found to have links with both social symptoms and the corpus callosum in the present study. To clarity brain-cognition-symptoms interactions, further structural equation model establishment would be in need.

Our study successfully established one mediation model, which indicated that fine motor function had a total mediating effect between the corpus callosum and perseveration symptoms in a combined sample of ASD and ADHD. Previous studies showed that the microstructure of the corpus callosum was associated with motor function [70], and fine motor impairment was increased in children with repetitive behavior [58]. In addition, Liu et al. found that fine motor skill training promoted corticospinal tract plasticity in rats with spinal cord injury [75]. The corpus callosum development affected the late stages of neurodevelopment in the fetus [76]. Axons of the neurons in the corpus callosum have been extensively pruned in the first 10 years of life [77], which is the onset time of neurodevelopment disorders. Therefore, we speculated that the abnormal remodeling of neurons represented by lower FA in the corpus callosum might cause fine motor dysfunction and further induce the appearance of perseveration symptoms. To the best of our knowledge, this was the first study on the brain structural connectivity-cognition-symptoms interaction model in ASD and ADHD, thus providing further evidence of a treatment target for neurodevelopmental disorders such as ASD and ADHD. Nonetheless, future studies with a larger sample size are warranted to verify reported findings.

This study had a few limitations. First, the sample size was relatively small, which might lead to overfitting. Thus, future studies with larger samples are in need to generate more subtle neurodevelopmental disorders subtypes. However, the validity of the clustering analysis had been validated to be robust in our data with parameters, such as, silhouette score, classification accuracy, adjusted Rand score and so on. Additionally, we also added neuroimaging and cognitive tests to validate the generated subtypes. Secondly, we included only high-functioning ASD (IQ > 70), which may not represent the full spectrum of ASD. The inclusion criteria were made to balance the IQ among groups, to eliminate the influence of intelligence on cognition and white matter structural connectivity profiles of symptom clusters as possible. Finally, there was a possibility of selection bias, given that the present study participants incorporated several selective cognition tests assessing a number of cognitive domains. Further subtype validation is needed, such as longitudinal follow-up of the prognosis of different subtypes, using broader neuroimaging methods and neurocognitive testing. In our next study, we plan to employ machine learning analysis to explore brain and/or neurocognitive measurements subtypes in a larger sample.

## Conclusion

This study demonstrated the effectiveness of a transdiagnostic approach for identifying neurodevelopmental subtypes incorporating ADHD with ASD. Three subtypes of symptom profiles were identified and validated across neurocognitive function and brain structural connectivity domains. Fine motor function and structural connectivity in the corpus callosum might be essential in distinguishing ASD-related from ADHD-related symptoms. A fine motor function had a mediating effect on the linkage between the structural connectivity of corpus callosum and perseveration symptoms. This study provided evidence for precise phenotyping by integrating behavioral and neurocognitive measurements as well as brain imaging data, thus promoting the exploration of new targeted interventions for neurodevelopmental disorders such as ASD and ADHD.

## Supplementary Information

**Additional file 1.**

## Data Availability

We guarantee the authenticity of the data, but do not disclose the data, if necessary, you can email huangyu@scu.edu.cn to obtain the data.

## Abbreviations

ASD: Autism Spectrum Disorders
ADHD: Attention Deficit Hyperactivity Disorder
ADI-R: Autism Diagnostic Interview-Revised
ADOS-G: Autism Diagnostic Observation Scale-General
K-SADS-PL: The schedule for affective disorders and schizophrenia for school-age children - Present and Lifetime Version
IQ: intelligence quotient
AQ: Autism-Spectrum Quotient
SNAP-IV: Swanson Nolan and Pelham, Version IV Scale
IA: SNAP-IV inattention subscale
HI: SNAP-IV hyperactivity/impulsivity subscale
ODD: SNAP-IV oppositional defiant disorder subscale
PPT: Purdue Pegbord Test
VP: Visual perception
MC: Motor coordination
VMI: Visual motor integration
VF-se: Semantic verbal fluency
VF-ph: Phoneny verbal fluency
EF: Executive function
RVP: Rapid visual information processing
SOC: Stockings of Cambridge
SWM: Spatial working memory
IED: Intra−/Extra-dimensional Set-shift Task
FA: Fractional anisotropy
