# Supplementary material for: Transdiagnostic symptom subtypes across autism spectrum disorders and attention deficit hyperactivity disorder: validated by measures of neurocognition and structural connectivity
Source: BMC Psychiatry. 2022 Feb 9;22:102. doi: 10.1186/s12888-022-03734-4 (PMC8827180; doi:10.1186/s12888-022-03734-4)
Supplement: Supplementary file 1 — Additional file 1. [file 12888_2022_3734_MOESM1_ESM.docx]

**Supplementary materials**

**Autism Diagnostic Interview-Revised (ADI-R) and Autism Diagnostic Observation Scale-General (ADOS-G)**

ADI-R is used for children aged from approximately 18 months to adulthood[1]. It is an investigator-based interview of guardians that covers most developmental and behavioral aspects of ASD, including reciprocal social interaction, communication, and repetitive behaviors and stereotyped patterns. ADOS-G is also a standardized behavioral observation and coding assessment for autism[2]. It has four different units for different levels of development and verbal abilities. ADI-R and ADOS-G are the golden standards for ASD diagnosis.

**The schedule for affective disorders and schizophrenia for school-age children - Present and Lifetime Version (K-SADS-PL)**

The schedule for affective disorders and schizophrenia for school-age children - Present and Lifetime Version (K-SADS-PL) is a semi-definite diagnostic scale based on the DSM-IV diagnostic criteria[3]. It is mainly used to assess the current and past psychopathic episodes of children and adolescents. Tests include emotional disorders, psychotic disorders, anxiety disorders, behavioral disorders, substance abuse, and other disorders. ADHD is a sub-item in behavioral disorders.

**Autism-Spectrum Quotient (AQ)**

The original AQ is a self-administered questionnaire for the explicit purpose of measuring the tendency towards autistic traits[4]. Cultural studies had been investigated in Chinese and showed good property (sensitivity 0.71, specificity 0.71) [5,6]. AQ consists of 35-item, 5-dimensional factors: socialness (little motivation in relating to others), mindreading (difficulty in perspective-taking or theory of mind), patterns, attention to details (exceptional attention to detail), attention switching (perseveration/strong focus of attention).

**Swanson Nolan and Pelham, Version IV Scale (SNAP-Ⅳ)**

The participants’ inattention and hyperactivity/impulsivity symptoms were assessed by the parent reports of the Chinese version of the Swanson, Nolan, and Pelham, version IV (SNAP-IV) scale. The scale is 4-Likert and consists of 26 items, 3 subscales: inattention, hyperactivity/impulsivity, and oppositional defiant disorder (ODD). The higher the score, the more severe the ADHD symptoms. Previous studies have shown good reliability and validity (discriminant accuracy 68.7%-75.1%)[7,8].

**Chinese-Wechsler Intelligence Scale for Children (C-WISC-III)**

Wechsler Intelligence Scales for Children-III (WISC-III)[9] was used to measure the intelligence of children. In this study, we used the short version of the Wechsler Children Intelligence Scale-III, which consists of vocabulary, similarity, picture completion, and block design.

**The Developmental Test of Visual-Motor Integration (Beery VMI)**

The Developmental Test of Visual-Motor Integration (Beery VMI)[10,11] is a developmental sequence of geometric forms to be reproduced with paper and pencil and scored according to objective scoring criteria outlined in the test manuals based on the imitation accuracy. The Beery VMI has been demonstrated to have good reliability and validity, as reported in the manual. Beery VMI is one of the most commonly used standardized measurements of visual-motor integration, visual perception, and motion coordination in several developmental disorders. The Beery VMI has a rich tradition in assessing children and adults with various neurodevelopmental disorders[12,13].

**The Purdue Pegboard Test (PPT)**

The Pegboard is equipped with pins, collars, and washers located in the proper cups[14]. The child is instructed to pick up one pin at a time with right/left/both hands and place these pins in the corresponding hand row, starting with the top hole. In the assembly sub-test, the testee should be allowed to assemble complete pin-washer-collar-washer assemblies. The five sub-test scores may be obtained with the Purdue Pegboard, namely: right hand, left hand, both hands, hands, and assembly. The results reflect the gross movements of hand, fingers, and arms as well as the ‘tip of the finger’ dexterity. The higher those scores are, the better the fine motor function they have.

**Verbal Fluency (VF)**

Verbal fluency included a phonemic fluency task and a semantic fluency task[15]. Phonemic fluency task require participants to generate words that correspond to a given production rule. In our study, the participant was asked to generate as many words as possible that begin with a particular letter ‘bo’. In a semantic fluency task, the participant is asked to generate as many words as possible that belong to a designated semantic category (e.g. animals). Participants have a given period of time to produce as many words as they can, typically in one minute.

**The Cambridge Neuropsychological Test Automated Battery (CANTAB)**

The Cambridge Neuropsychological Test Automated Battery CANTAB eclipse Test (Administration Guide Manual, <http://www.cambridgecognition.com/>) is a set of computerized paradigms running on a computer with a high-resolution colofffr monitor and the touch-sensitive screen. We chosed Rapid visual information processing (RVP), Stockings of Cambridge (SOC), Spatial Working Memory (SWM), Intra-/Extra-dimensional Set-shift Task (IED) subtests in our study.

**Rapid visual information processing (RVP)**

Subjects are requested to detect target sequences of digits (1-2-3) to register responses using the press pad. RVP A is the outcome measure for how good the subject is at detecting target sequences, which ranges from 0.00 to 1.00. The higher the scores are, the better the attention is.

**Stockings of Cambridge (SOC)**

The subject must use the balls in the lower display to copy the pattern shown in the upper display. The problems solved in minimum moves is a fundamental measure, recording the number of occasions upon which the subject has completed a test problem in the minimum possible number of moves. The higher scores are, the better is planning.

**Spatial Working Memory (SWM)**

Kids were asked to search through several colored boxes presented on the screen to find blue tokens hidden inside, which is set to be shown only once a trail in each box. The outcome for the SWM test may be regarded as “Total Errors” in previous studies. The lower the total errors of SWM, the better the cognitive function of the Spatial Working Memory.

**Intra-/Extra-dimensional Set-shift Task (IED)**

Subjects were presented with two pictures, and they must learn which of the stimulus is correct by touching it, and continue until the criteria are reached. Then the contingencies are reversed so that now the previously incorrect stimulus is correct. The subjects need to learn to adapt to the changing rules. IED total errors (adjusted) is a measure of the subject’s efficiency in attempting the test. Lower reflect better flexibility.

**Supplementary Tables and Figures**

Table S1. Differences Between Clusters on Profiles of Symptom Severity

Table S2. Discriminant Function Loadings and composites differential effect for clusters

Table S3. Percentage of children with each diagnosis within clusters

Figure S1. Symptom scores PCA Plot for Three subtypes

**Table S1. Differences Between Clusters on Profiles of Symptom Severity.**

|  | Cluster 1 | Cluster 2 | Cluster 3 | F | p | Post-hoc |
| --- | --- | --- | --- | --- | --- | --- |
| Age | 8.92 (3.21) | 8.71(2.76) | 8.92 (2.56) | 0.10 | 0.9073 | - |
| IQ | 98.57(18.07) | 101.99(17.02) | 105.82(18.66) | 2.15 | 0.1203 | - |
| Sex（W/M） | 59/6 | 47/8 | 33/9 | 4.302 | 0.1164 | - |
| AQ | 58.85(9.27) | 40.31(11.32) | 32.67(8.86) | 103.46 | <0.001*** | C3<C2<C1 |
| SNAP-Ⅳ | 39.30(8.21) | 24.80(7.39) | 44.17(8.18) | 82.64 | <0.001*** | C2<C1<C3 |
| Socialness | 20.16 (4.97) | 13.15(4.88) | 7.21 (3.36) | 106.60 | <0.001*** | C3<C2<C1 |
| Mindreading | 14.81 (4.00) | 7.80(4.81) | 6.48 (4.30) | 60.99 | <0.001*** | C3<C2<C1 |
| Patterns | 6.69 (2.77) | 5.80(2.76) | 5.83 (2.24) | 2.16 | 0.1184 | - |
| Details | 6.21 (2.40) | 5.49(2.60) | 5.88 (2.18) | 1.33 | 0.2661 | - |
| Perseveration | 10.99 (2.86) | 8.07(2.91) | 7.26 (2.72) | 27.09 | <0.001*** | C3<C2<C1 |
| IA | 15.81 (4.31) | 12.53 (4.70) | 17.31(3.57) | 16.47 | <0.001*** | C2<C1/C3 |
| HI | 12.99 (4.22) | 6.69(3.40) | 14.69(3.88) | 61.31 | <0.001*** | C2<C1/C3 |
| ODD | 10.51 (4.13) | 5.58(3.45) | 12.17(4.49) | 37.31 | <0.001*** | C2<C1/C3 |

Note: One-Way ANOVA with Bonferroni post-hoc test. ***p < 0.001, IQ: intelligence quotient. AQ: Autism-Spectrum Quotient total scores; SNAP-Ⅳ: Swanson Nolan and Pelham, Version IV Scale total scores; socialness: AQ socialness subscale, mindreading: AQ mindreading subscale, patterns: AQ patterns subscale, details: AQ attention to details, perseveration: AQ attention switching/perseveration subscale. IA: SNAP-Ⅳ inattention subscale, HI: SNAP-Ⅳ hyperactivity/impulsivity, ODD: SNAP-Ⅳ oppositional defiant disorder subscale.

**Table S2. Discriminant Function Loadings and composites differential effect for clusters**

| symptoms | Composites | | Lambda | Discriminant Function |
| --- | --- | --- | --- | --- |
|  | DF1 | DF2 |  |  |
| DF1 |  |  |  | 0.797*** |
| socialness | **0.818** | -0.388 | 0.281*** |  |
| mindreading | **0.659** | -0.034 | 0.249*** |  |
| perseveration ^b^ | **0.409** | -0.154 | 0.177 |  |
| details ^b^ | **0.053** | 0.013 | 0.170 |  |
| DF2 |  |  |  | 0.716*** |
| HI | 0.217 | **0.805** | 0.211*** |  |
| ODD | 0.148 | **0.637** | 0.202*** |  |
| IA ^b^ | 0.108 | **0.147** | 0.193 |  |
| patterns ^b^ | 0.023 | **-0.071** | 0.176 |  |

Note: *p < 0.05, **p < 0.01, ***p < 0.001; socialness: AQ socialness subscale, mindreading: AQ mindreading subscale, patterns: AQ patterns subscale, details: AQ attention to details, perseveration: AQ attention switching/perseveration subscale. IA: SNAP-Ⅳ inattention subscale, HI: SNAP-Ⅳ hyperactivity/impulsivity, ODD: SNAP-Ⅳ oppositional defiant disorder subscale. The Linear discriminant analysis constructs a predictive model to evaluate group membership, these new variables are called discriminants functions (DF’s) and based on linear combinations of the predictive variables that provide the best discrimination between the groups; ^b^ the variable was excluded in the model. The loadings of the symptoms composite variables on the two discriminant functions are shown under the ‘Composites’ column.

**Table S3. Percentage of children with each diagnosis within clusters**

| Diagnosis | Cluster 1 | | Cluster 2 | | Cluster 3 | | x2 | p |
| --- | --- | --- | --- | --- | --- | --- | --- | --- |
|  | number | percent | number | percent | number | percent |  |  |
| ASD | 41 | 61.19% | 19 | 34.55% | 5 | 11.90% |  |  |
| ADHD | 16 | 23.88% | 7 | 12.73% | 24 | 57.14% |  |  |
| TDC | 10 | 14.93% | 29 | 52.72% | 13 | 30.96% |  |  |
| Total | 67 | - | 55 | - | 42 | - | 47.28 | <0.001 |

Note: ASD: autism spectrum disorder; ADHD: attention deficient hyperactivity disorder; TDC: typically developed control


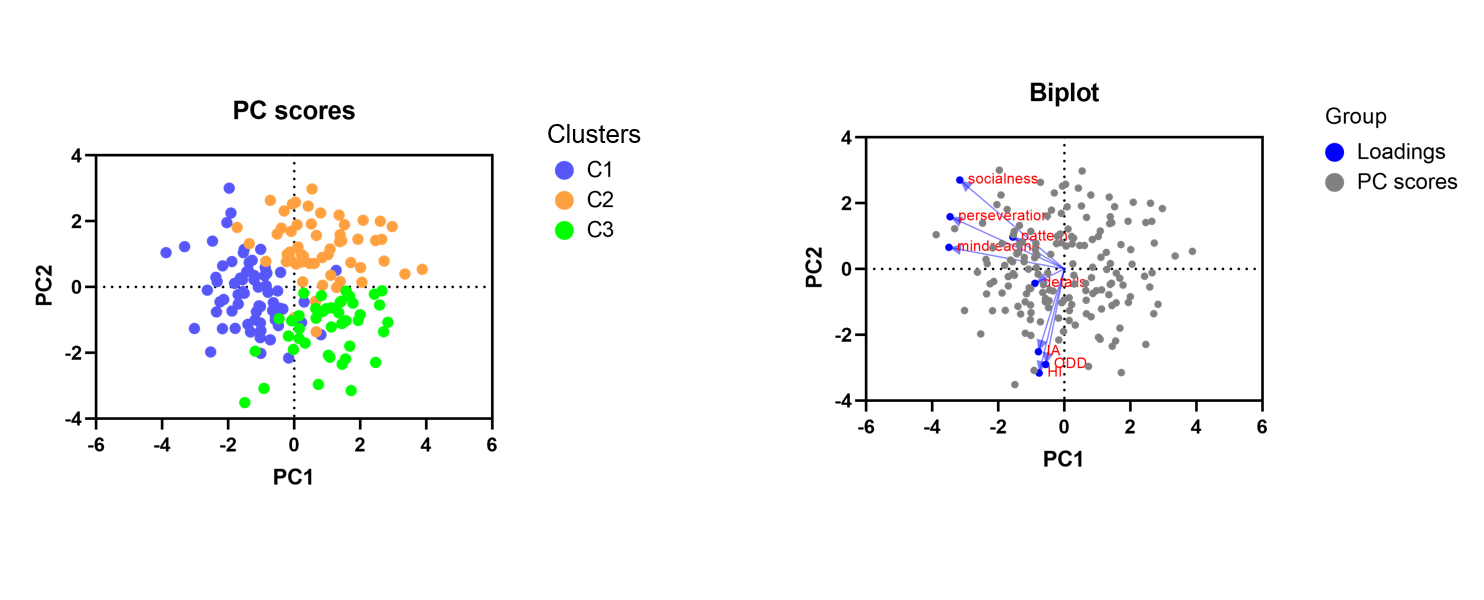


**Figure S1. Symptom scores PCA plot for three subtypes**

This left figure showed all samples projected across the first two components of a principal components analysis (PCA), calculated using the symptoms data. This plot colors samples based on the three symptom subtypes. The right figure demonstrated the loadings of the eight dimensions.

**References**

1. Lord C, Rutter M, Couteur AL (1994) Autism Diagnostic Interview-Revised: A revised version of a diagnostic interview for caregivers of individuals with possible pervasive developmental disorders. Journal of Autism & Developmental Disorders 24 (5):659-685. doi:10.1007/BF02172145

2. Lord C, Risi S, Lambrecht L, Cook EH, Leventhal BL, Dilavore PC, Pickles A, Rutter M (2000) The Autism Diagnostic Observation Schedule-Generic: A Standard Measure of Social and Communication Deficits Associated with the Spectrum of Autism. J Autism Dev Disord 30 (3):205-223

3. Monk CS, Peltier SJ, Wiggins JL, Weng SJ, Carrasco M, Risi S, Lord C (2009) Abnormalities of intrinsic functional connectivity in autism spectrum disorders. Neuroimage 47 (2):764-772. doi:10.1016/j.neuroimage.2009.04.069

4. Baron-Cohen S, Wheelwright. S, Skinner. R, Martin. J, Clubley. E (2001) The Autism-Spectrum Quotient (AQ): Evidence from Asperger Syndrome/High-Functioning Autism, Malesand Females, Scientists and Mathematicians. J Autism Dev Disord 31 (1):5-17. doi:10.1023/A:1005653411471

5. Lau YP, Gau SS-F, Chiu Y-N, Wu Y-Y, Chou W-J, Liu S-K, Chou M-C Psychometric properties of the Chinese version of the Autism Spectrum Quotient (AQ). Research in Developmental Disabilities 34 (1):294-305. doi:10.1016/j.ridd.2012.08.005

6. Zhang MX, Yang PY, Hu X, Guo KF, Situ MJ, Huang Y (2018) [Reliability and Validity of the Abridged Chinese Version of Autism Spectrum Quotient-Child form in China]. Sichuan da xue xue bao Yi xue ban = Journal of Sichuan University Medical science edition 49 (4):599-603

7. Swanson JM, Kraemer HC, Hinshaw SP, Arnold LE, Conners CK, Abikoff HB, Clevenger W, Davies M, Elliott GR, Greenhill LL, Hechtman L, Hoza B, Jensen PS, March JS, Newcorn JH, Owens EB, Pelham WE, Schiller E, Severe JB, Simpson S, Vitiello B, Wells K, Wigal T, Wu M (2001) Clinical relevance of the primary findings of the MTA: success rates based on severity of ADHD and ODD symptoms at the end of treatment. J Am Acad Child Adolesc Psychiatry 40 (2):168-179. doi:10.1097/00004583-200102000-00011

8. Gau. SS-F, Shang. C-Y, Liu. S-K, Lin. C-H, Tu. C-L (2008) Psychometric properties of the Chinese version of the Swanson, Nolan, and Pelham, Version IV Scale-Parent Form. International Journal of Methods in Psychiatric Research 17 (1):35-44. doi:10.1002/mpr.237

9. Woolger C (1995) Wechsler Intelligence Scale for Children-Third Edition (wisc-iii). 28 (4):219-224

10. Spencer. TD, Kruse. L (2013) Beery-Buktenica Developmental Test of Visual-Motor Integration. doi:10.1007/978-1-4419-1698-3_1886

11. Spencer TD, Kruse L Beery-Buktenica Developmental Test of Visual-Motor Integration.

12. Michael H Bloch DGS, Philip A Dombrowski, Kaitlyn E Panza, Brittany G Craiglow, Angeli Landeros-Weisenberger, James F Leckman, Bradley S Peterson, Robert T Schultz (2011) Poor fine-motor and visuospatial skills predict persistence of pediatric-onset obsessive-compulsive disorder into adulthood. Journal of Child Psychology & Psychiatry 52 (9):974-983

13. Green RR, Bigler ED, Froehlich A, Prigge MBD, Travers BG, Cariello AN, Anderson JS, Zielinski BA, Alexander A, Lange N (2016) Beery VMI performance in autism spectrum disorder.

14. Tiffin J, Asher EJ (1948) The Purdue pegboard; norms and studies of reliability and validity. The Journal of applied psychology 32 (3):234-247. doi:10.1037/h0061266

15. Sandy Harth SV, Müller S, Aschenbrenner O, Tucha KW, Lange (2004) Regensburger wortflüssigkeits-test(rwt). Ztschrift für Neuropsychologie 15 (4):315-321
